# Coupling one-dimensional arterial blood flow to three-dimensional tissue perfusion models for *in silico* trials of acute ischaemic stroke

**DOI:** 10.1098/rsfs.2019.0125

**Published:** 2020-12-11

**Authors:** Raymond M. Padmos, Tamás I. Józsa, Wahbi K. El-Bouri, Praneeta R. Konduri, Stephen J. Payne, Alfons G. Hoekstra

**Affiliations:** 1 Computational Science Laboratory, Institute for Informatics, Faculty of Science, University of Amsterdam, Science Park 904, 1098 XH Amsterdam, The Netherlands; 2 Institute of Biomedical Engineering, Department of Engineering Science, University of Oxford, Parks Road, Oxford OX1 3PJ, UK; 3 Department of Radiology and Nuclear Medicine, Amsterdam UMC, Location AMC, 1105 AZ Amsterdam, The Netherlands; 4 Biomedical Engineering and Physics, Amsterdam UMC, Location AMC, 1105 AZ Amsterdam, The Netherlands

**Keywords:** one-dimensional blood flow model, perfusion territories, cerebral perfusion, acute ischaemic stroke

## Abstract

An acute ischaemic stroke is due to the sudden blockage of an intracranial blood vessel by an embolized thrombus. In the context of setting up *in silico* trials for the treatment of acute ischaemic stroke, the effect of a stroke on perfusion and metabolism of brain tissue should be modelled to predict final infarcted brain tissue. This requires coupling of blood flow and tissue perfusion models. A one-dimensional intracranial blood flow model and a method to couple this to a brain tissue perfusion model for patient-specific simulations is presented. Image-based patient-specific data on the anatomy of the circle of Willis are combined with literature data and models for vessel anatomy not visible in the images, to create an extended model for each patient from the larger vessels down to the pial surface. The coupling between arterial blood flow and tissue perfusion occurs at the pial surface through the estimation of perfusion territories. The coupling method is able to accurately estimate perfusion territories. Finally, we argue that blood flow can be approximated as steady-state flow at the interface between arterial blood flow and tissue perfusion to reduce the cost of organ-scale simulations.

## 1. Introduction

An acute ischaemic stroke is the most common type of stroke. It is fatal to an estimated 3 million people globally every year and may lead to a dramatic loss of quality of life in survivors [1]. An acute ischaemic stroke occurs when blood supply to the brain is suddenly blocked by an embolized thrombus. This results in the acute loss of blood flow to downstream vessels. Consequently, brain tissue is starved of oxygen, resulting in necrosis, causing disability and ultimately death. Currently, only a few treatment options exist. Improving existing treatments and developing and approving new treatments is a difficult, lengthy and expensive process, prone to failures during clinical trials even after successful animal trials.

The INSIST project^1^ [2,3] aims to develop computational models that will be used to simulate a clinical stroke trial on a virtual population. The (virtual) patient in this context is generally a set of parameters and other data that are used in a computational model. *In silico* trials (IST) can overcome some of the limitations of clinical trials, lower their cost, contribute to developing more efficient clinical trials or shorten the preclinical trajectories [4]. IST are closely related to computational biomedicine or personalized medicine. The goal is to predict the efficacy and efficiency of a treatment, drug or device. As such, they are similar to normal clinical trials where the testing happens on animals or humans. Computational modelling has become the standard in many industries from the design of air planes to electronics. Likewise, computational models in the medical field are slowly gaining momentum [5–10].

One of the goals of INSIST is to predict the location and volume of the infarcted brain tissue for stroke patients both for individual patients and at the population level. This requires modelling blood flow across length scales incorporating three orders of magnitude, from the large arteries via the arterioles and the pial surface vessels to the penetrating vessels and the microcirculation deep in the brain. Blood flow in large vessels is typically modelled using lumped parameter or one-dimensional (1D) blood flow models, whereas the microcirculation is typically modelled as a porous medium [11]. In addition, there is limited knowledge about the vessel anatomy between the large vasculature and the microcirculation. Data on the intermediate vessels are limited or only available as statistical scaling laws. Predicting infarcted volume requires thus the coupling of arterial blood flow models to tissue perfusion models. Inclusion of the collateral circulation is also important but is outside the scope of this paper. A tissue perfusion model is presented in another article in this same special issue of *Interface Focus* [12]. A 1D blood flow model has to be coupled to this tissue perfusion model, which presents several challenges.

Firstly, medical images often suffer from low resolution. Scans from stroke patients are often done quickly to get an assessment of the stroke severity. As a result, the scans typically only contain the circle of Willis (CoW) and a few of the major cerebral arteries. This presents a problem as this does not provide sufficient information for the coupling between arterial blood flow and tissue perfusion. However, there are high-resolution scans available from different individuals [13,14]. Unfortunately, this dataset, referred to as the BraVa dataset, does not contain other information such as, for example, pressure, heart rate and Young's modulus.

Secondly, information about the perfusion territories of the cerebral arteries can be determined but is limited [15]. The perfusion territory of a blood vessel is the region of tissue that receives blood from that vessel. Not much is known about the perfusion territories of the brain and data are again sparse. Coupling blood flow to tissue perfusion in a patient-specific manner requires the estimation of the perfusion territory for every perfusing vessel.

Here, a method is presented to couple blood flow in cerebral blood vessels to cerebral tissue perfusion. Patient-specific image-based vessel segmentations are combined with literature data and models for vessel anatomy not visible in the images to create an extended model for each patient. Using Murray's Law, we estimate perfusion territories on the pial surface using a coupling algorithm. Arterial blood flow is simulated with a 1D pulsatile blood flow model with three-element windkessel elements at the boundaries. We show that, at the pial surface, blood flow pulsatility is small and argue that blood flow can be approximated as steady-state flow.

## 2. Methods

### 2.1. Vasculature of virtual patients

**Figure 1.**
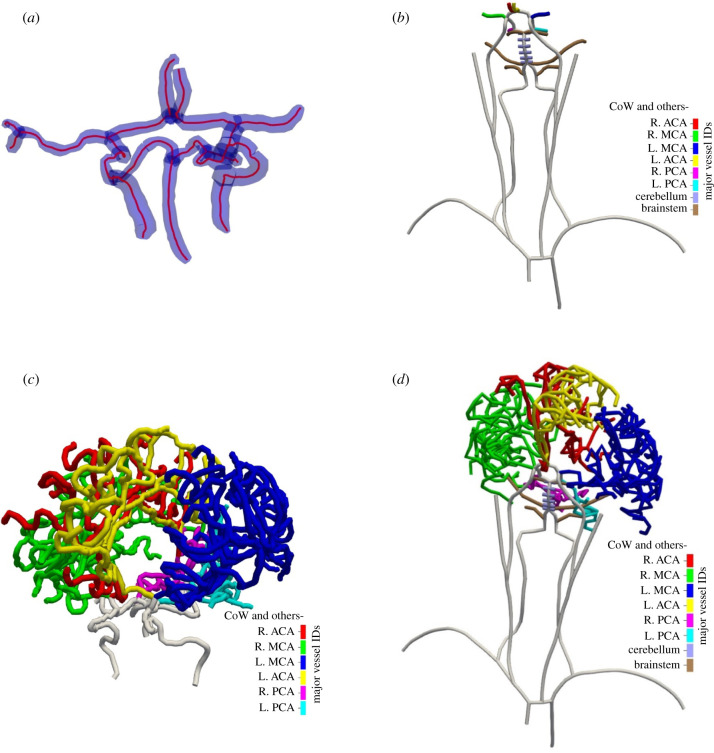
(*a*) Segmentation from a CT angiography from a stroke patient in blue. The segmentation contains the circle of Willis and several side branches. Centrelines are extracted and shown in red. Circular cross-sections are used as estimates for the radius. (*b*) The large arteries, starting from the heart leading up to and including the patient-specific CoW. Smaller vessels to the brainstem and cerebellum are also included. Note that the vessels are not drawn to scale. (*c*) One of the centreline segmentations from the BraVa dataset (ID: BG18). Each vessel is smoothed by setting the radius at every point to the mean radius of that vessel. Vessels are colour coded according to which major cerebral vessel they belong. The CoW vessels from the BraVa segmentation are not used and are replaced by the patient-specific CoW from (*a*). (*d*) The final patient-specific arterial network. Patient-specific data from (*a*) are extended with literature values from (*b*) and high-resolution data from (*c*). This is the network that is used in the 1D blood flow simulations. Note that the vessels are not drawn to scale.

Vessel centrelines are extracted from vessel segmentations of computed tomography (CT) angiographs from stroke patients entering the hospital [16].^2^ The centrelines contain the CoW and the side branches, figure 1*a*. The side branches are the posterior cerebral artery (PCA), anterior cerebral artery (ACA) and middle cerebral artery (MCA). This patient-specific vasculature is first extended with the larger vessels, starting from the heart, figure 1*b*. The smaller vessels to the cerebellum and brainstem are also added. The default parameters describing these arteries are taken from the literature [17,18] and are shown in table 1. A taper can be included by setting different proximal and distal radii but has not been considered in this paper.

**Table 1.** Default values for large vessels, adapted from [16,18].

vessel name	length (mm)	proximal radius (mm)	distal radius (mm)	Young's modulus (MPa)
ascending aorta	40	12	12	0.4
aortic arch I	20	11.2	11.2	0.4
brachiocephalic	34	6.2	6.2	0.4
aortic arch II	39	11	11	0.4
L. common carotid	208	2.5	2.5	0.4
R. common carotid	177	2.5	2.5	0.4
R. subclavian	34	4.23	4.23	0.4
thoracic aorta	156	9.99	9.99	0.4
L. subclavian	34	4.23	4.23	0.4
L ext. carotid	177	1.5	1.5	0.8
L int. carotid I	177	2	2	0.8
R int. carotid I	177	2	2	0.8
R ext. carotid	177	1.5	1.5	0.8
R. vertebral	148	1.36	1.36	0.8
R. brachial	422	4.03	4.03	0.4
L. brachial	422	4.03	4.03	0.4
L. vertebral	148	1.36	1.36	0.8
L. PCoA	15	0.73	0.73	1.6
R. PCoA	15	0.73	0.73	1.6
basilar I	5.6	1.62	1.62	1.6
L. MCA	119	1.43	1.43	1.6
R. MCA	119	1.43	1.43	1.6
L. ACA, A1	12	1.17	1.17	1.6
R. ACA, A1	12	1.17	1.17	1.6
L. PCA, P1	5	1.07	1.07	1.6
R. PCA, P1	5	1.07	1.07	1.6
L. ACA, A2	103	1.2	1.2	1.6
R. ACA, A2	103	1.2	1.2	1.6
ACoA	3	0.74	0.74	1.6
L. PCA, P2	86	1.05	1.05	1.6
R. PCA, P2	86	1.05	1.05	1.6
R. SCA	10	0.78	0.78	1.6
L. SCA	10	0.78	0.78	1.6
R. AICA	10	0.63	0.63	1.6
L. AICA	10	0.63	0.63	1.6
basilar II	5.6	1.62	1.62	1.6
pontine I	5	0.2	0.2	1.6
pontine II	5	0.2	0.2	1.6
pontine III	5	0.2	0.2	1.6
pontine IV	5	0.2	0.2	1.6
pontine V	5	0.2	0.2	1.6
pontine VI	5	0.2	0.2	1.6
pontine VII	5	0.2	0.2	1.6
pontine VIII	5	0.2	0.2	1.6
pontine IX	5	0.2	0.2	1.6
pontine X	5	0.2	0.2	1.6
pontine XI	5	0.2	0.2	1.6
pontine XII	5	0.2	0.2	1.6
basilar III	5.6	1.62	1.62	1.6
basilar IV	5.6	1.62	1.62	1.6
basilar V	5.6	1.62	1.62	1.6
R. PICA	10	0.63	0.63	1.6
L. PICA	10	0.63	0.63	1.6
R. vertebral II	20	1.36	1.36	0.8
L. vertebral II	20	1.36	1.36	0.8

Centrelines of high-resolution scans of cerebral arteries are available from 61 different individuals [13,14]. These centrelines are referred to as the BraVa dataset. The arteries in the datasets are labelled by six major cerebral arteries from which they emerged, i.e. left and right ACA, MCA and PCA, and the CoW, figure 1*c*. One segmentation of the BraVa set is randomly chosen and attached to the patient-specific vasculature. For each major cerebral artery in the patient vasculature, the equivalent vessel in the BraVa vasculature is used as an attachment point. The radii of the added vessels are scaled by the ratio of equivalent cerebral vessels, ACA, MCA, PCA, etc., between the BraVa dataset and the patient-specific vasculature. All radii are scaled to preserve any scaling law between the vessels.

These three sets of data are combined to create a patient-specific vasculature that starts at the heart and ends close to the pial surface. For each major cerebral artery in the patient dataset, the relevant vessels in the Brava dataset are identified and then attached to each other. On the other end of the patient anatomy, the large arteries to the heart are connected, as shown in figure 1*d*.

### 2.2. One-dimensional blood flow modelling

One-dimensional blood flow models assembled from multiple vessel segments [19–25] are able to model blood flow in sufficient detail without becoming too computationally expensive. These 1D models capture the axial flow component along the vessel, showing a good agreement with 3D blood flow simulations [26]. Blood flow is modelled as a viscous incompressible fluid. Assuming rotational symmetry around the centreline of the circular vessels and integrating the continuity equation along the azimuthal and the radial coordinates of a cylindrical coordinate system [19,22] leads to

2.1 $$\begin{array}{c} \frac{\partial A}{\partial t}+\frac{\partial (A{\bar{v}}_{x})}{\partial x}=0. \end{array}$$

Following the same steps results in the averaged Navier–Stokes momentum equations

2.2 $$\begin{array}{c} \frac{\partial {\bar{v}}_{x}}{\partial t}+(2\alpha -1){\bar{v}}_{x}\frac{\partial {\bar{v}}_{x}}{\partial x}+(\alpha -1)\frac{{\bar{v}}_{x}^{2}}{A}\frac{\partial A}{\partial x}+\frac{1}{\rho }\frac{\partial p}{\partial x}=\frac{-2\alpha \mu \pi {\bar{v}}_{x}}{(\alpha -1)\rho A}. \end{array}$$

The primary variables are the cross-sectional area of the vessel (*A*), the averaged axial velocity along the vessel (${\bar{v}}_{x}$) and static pressure (*p*) within each vessel as functions of the axial coordinate (*x*) and time (*t*). The corresponding model parameters are the momentum correction factor (*α*), the density (*ρ*) and the dynamic viscosity of blood (*μ*). A stroke is modelled as a complete blockage of flow and pressure by enforcing Neumann pressure boundary conditions at the end of the clotted vessel segment. In the future, the model will be extended to handle partial occlusions.

In order to close the equation system formed by equations (2.1) and (2.2), a relationship between the pressure and the cross-sectional area has to be defined. Wall deformations are described by a thin-walled axisymmetric elastic membrane approach so that

2.3 $$\begin{array}{c} \;p=p_{\mathrm{ref}}+\frac{\beta }{A_{0}}\left(\sqrt{A}-\sqrt{A_{0}}\right), \end{array}$$

where

2.4 $$\begin{array}{c} \beta =\frac{\sqrt{\pi }Eh}{\left(1-\nu^{2}\right)}. \end{array}$$

Here, *A*_0_ is the area of the lumen at *p*_ref_ and *p*_ref_ is a reference pressure set to the diastolic pressure. The *β* coefficient is determined by the vessel wall properties, which are constant in each segment, including the wall thickness (*h*), the Poisson's ratio (ν) and the Young's modulus (*E*) of the vessel wall (constants within vessel segments). The thickness of the vessel wall is calculated using an empirical function given by

2.5 $$\begin{array}{c} h=r_{0}[a\exp(br_{0})+c\exp(dr_{0})], \end{array}$$

with *r*_0_ the initial radius, *a* = 0.2802, *b* = −0.5053 mm^−1^, *c* = 0.1324 and *d* = −0.01114 mm^−1^ [25].

The method used here to solve the resulting equations is the MacCormack method, a second-order finite difference method. The initial conditions are zero velocity and diastolic pressure everywhere. Vessels are linked together at the bifurcations by the method of characteristics for continuous propagation of characteristics, conservation of mass and total continuity of pressure (Bernoulli's equation) [19–25]. The resulting equations are solved with the Newton–Raphson method.

Every vessel has a minimum of three nodes and a maximum separation of 10 mm. The simulation is run for a small number of heart beats and iterated until the tolerance between iterations is below a threshold, defined as

2.6 $$\begin{array}{c} \frac{\left|p_{i}-p_{i-1}\right|}{\left|p_{i}\right|}<10^{-3}\;, \end{array}$$

with $p_{i}$ being a vector of the pressure at all *n* nodes at all *m* timesteps during the *i*th cardiac cycle and $p_{i-1}$ a vector of the pressure at the previous iteration *i* − 1. That is $p_{i}=[\;p_{1}(t_{1}),p_{1}(t_{2})\ldots p_{n}(t_{m})]$. The typical number of simulated cardiac cycles before convergence is below 10.

The heart provides the inlet boundary conditions to the model while a three-element windkessel model is used at each outlet. Note that the BraVa anatomies typically have of the order of 100 outlets. Volume flow rate at the inlet is given by an inlet function and taken from Boileau *et al.* [23]. The inlet function is scaled based on stroke volume and heart rate for each patient. The total resistance (*R*_tot_) and compliance (*C*_tot_) of the full vascular tree model are calculated by [27]

2.7 $$\begin{array}{c} R_{\mathrm{tot}}=\frac{(1/3)P_{\mathrm{sys}}+(2/3)P_{\mathrm{dia}}}{\text{SV}\cdot \text{HR}} \end{array}$$

and

2.8 $$\begin{array}{c} C_{\mathrm{tot}}=\frac{\tau }{R_{\mathrm{tot}}}, \end{array}$$

with *P*_sys_ the systolic pressure, *P*_dia_ the diastolic pressure, SV the stroke volume, HR the heart rate and *τ* the aortic pressure decay time constant. The compliance of the patient network (*C*_1D_) is calculated from the sum of the vessel compliances given by

2.9 $$\begin{array}{c} C_{1D}=\frac{3A\sqrt{A}L}{2\sqrt{\pi }Eh}, \end{array}$$

where *L* is the vessel length. The network compliance is substantial for a large vessel and is, therefore, subtracted from the total compliance. The resistance of the patient network is assumed to be negligible for the large arteries compared to the total resistance.

Known distributions of cardiac output into the main vessels are used to distribute the resistance and compliance to each outlet. That is, 65% goes to the lower body through the thoracic aorta, 5% goes to the arms through the left and right brachial arteries and the rest to the other outlets [28]. The resistance and compliance are distributed to the outlets, *R*_*T*_ and *C*, based on the fraction of the radius cubed, e.g. Murray's Law [29]. The cardiac output distribution is assumed to be constant. The total resistance and compliance for an outlet are given by

2.10 $$\begin{array}{c} R_{T}=\frac{R_{\mathrm{tot}}}{\text{C}{\text{O}}_{f}}\frac{\sum_{i}r_{0i}}{r_{0}} \end{array}$$

and

2.11 $$\begin{array}{c} C=\left(C_{\mathrm{tot}}-\sum_{\mathrm{vessels}}{\text{C}}_{1D}\right)\frac{R_{\mathrm{tot}}}{R_{T}}, \end{array}$$

where *i* sums over all outlets connected to a particular body part and CO*_f_* is the cardiac output fraction of that body part.

The total resistance of the attached cerebral arteries is subtracted to account for the resistance added by the attachment of the BraVa vessels to the patient network. The resistance of the added vasculature is calculated by iteratively adding resistances depending on whether vessels run in parallel or serial. The two resistance windkessel parameters, *R*_1_ and *R*_2_, of each outlet are calculated by

2.12 $$\begin{array}{c} R_{1}=\frac{\rho }{A_{0}}\sqrt{\frac{2Eh}{3\rho r_{0}}} \end{array}$$

and

2.13 $$\begin{array}{c} R_{2}=R_{T}-R_{1}. \end{array}$$

The choice for *R*_1_ leads to the minimization of unnatural wave reflections at the outlets [30]. The outlets of the 1D blood flow model provide blood flow to the perfusion territories of the brain. The default parameters of the model are listed in table 2.

**Table 2.** Default model parameters.

parameter	symbol	value
density	*ρ*	1050 kg m^−3^
viscosity	*μ*	0.0035 Pa s
venous pressure		2500 Pa
heart rate	HR	60 min^−1^
systole pressure	*P*_sys_	17 300 Pa
diastole pressure	*P*_dia_	10 100 Pa
stroke volume	SV	70 ml
momentum correction factor	*α*	1.1
aortic pressure decay time	*τ*	1.34 s
Poisson ratio	*ν*	0.5

The extension process proceeds along the following steps:

(1) identify identical vessels in both datasets by their label;
(2) extract the smaller upstream vessels starting from the major cerebral artery;
(3) calculate the mean radius for identical vessels in both datasets;
(4) scale the radii of the upstream vessels identified previously accordingly;
(5) attach the upstream vessels to the relevant end in the patient vasculature;
(6) calculate the resistance of the appended vessels;
(7) subtract the resistance and distribute the remainder to the appended ends.

To characterize the strength of the pulsatility of blood flow, pulsatility indices are used. The pressure pulsatility index is given by

2.14 $$\begin{array}{c} Pi_{p}=\frac{\;p_{\max}-p_{\min}}{\;p_{\mathrm{mean}}}, \end{array}$$

with *p*_max_ the maximum pressure, *p*_min_ the minimum pressure and *p*_mean_ the mean pressure over a single heartbeat. The velocity pulsatility index is given by

2.15 $$\begin{array}{c} Pi_{v}=\frac{v_{\max}-v_{\min}}{v_{\mathrm{mean}}}, \end{array}$$

with *v*_max_ the maximum velocity, *v*_min_ the minimum velocity and *v*_mean_ the mean velocity.

### 2.3. Estimating perfusion territories

Coupling blood flow to tissue perfusion in a patient-specific manner requires the estimation of the perfusion territory for every perfusing vessel, so for every outlet of the BraVa sets (i.e. left and right ACA, MCA and PCA), the cerebellum and the brainstem. Using Murray's Law, perfusion territories on the pial surface are estimated using a coupling algorithm. The radius of the outlet is assumed to be related to the perfusion of tissue by Murray's Law given by

2.16 $$\begin{array}{c} r_{i}^{3}\propto Q_{i}, \end{array}$$

with $r_{i}^{3}$ the cubic radius at outlet *i* and *Q_i_* the volume flow rate at outlet *i*.

We estimate the fraction of the pial surface that belongs to each outlet *i* of the patient vasculature at the pial surface with the use of Murray's Law. This fraction is calculated as

2.17 $$\begin{array}{c} \;f_{i}=\frac{Q_{i}}{\sum_{\;j=0}^{n}Q_{j}}=\frac{r_{i}^{3}}{\sum_{\;j=0}^{n}r_{j}^{3}}, \end{array}$$

with *Q_i_* the volume flow rate at the outlet and $r_{i}^{3}$ the cubic radius at the outlet. The proportionality constants from equation (2.16) drop out in equation (2.17) because they are set to be the same at all outlets. The volume flow rate per area, or flow velocity, is uniform over the pial surface under these assumptions. To what extent this is also true for a real brain is not clear.

**Figure 2.**
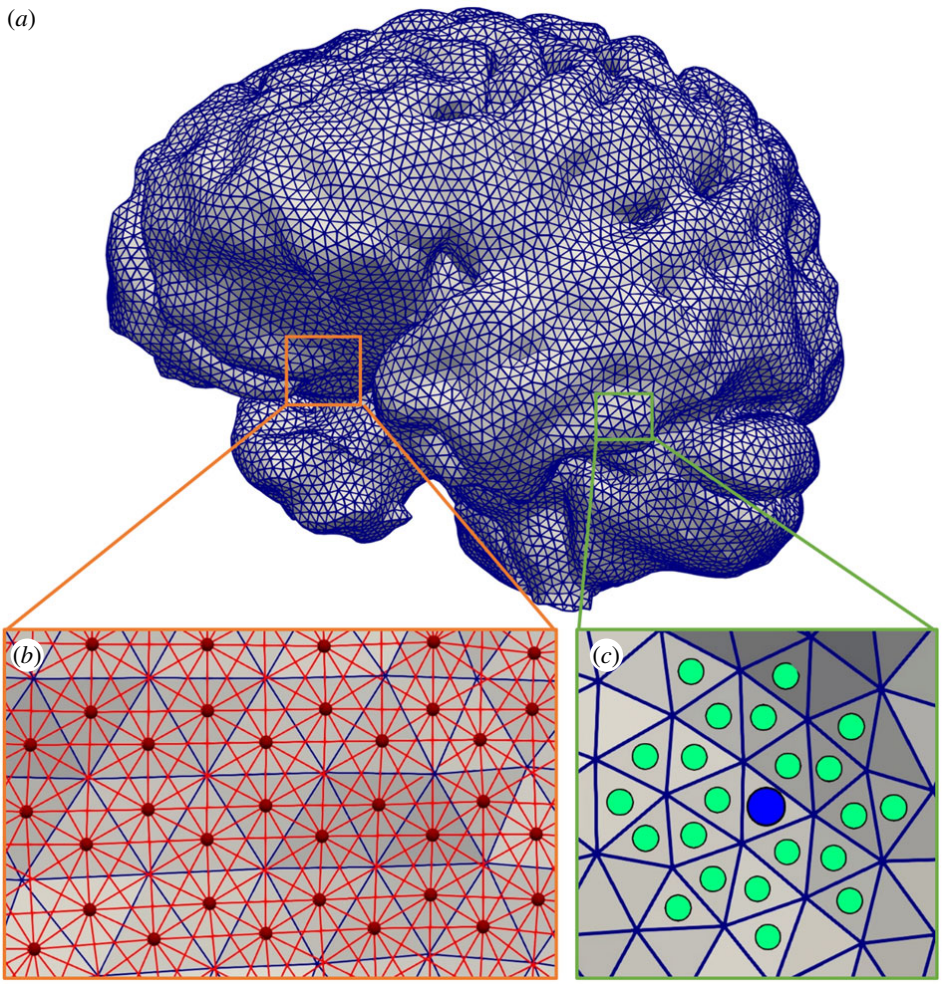
(*a*) The pial surface as a uniform triangulated mesh. (*b*) Nodes are the centres of the triangles from (*a*), shown as red circles, and connected by red lines if they share a vertex. The distance between connected nodes is the Euclidean distance. (*c*) The outlet of a vessel is projected to the surface, blue circle. The nearest surface elements, i.e. triangles, are assigned to the nearest outlet, green circles. Distance between surface elements is determined by Dijkstra's algorithm from the graph in (*b*).

The pial surface is represented as a uniform triangulated mesh, figure 2*a*, where each triangle represents an equal sized area on the mesh. The mesh was presented in the study of Garcia-Gonzalez *et al.* [31] where it was used for finite-element computations. The triangles are grouped based on distance, calculated with Dijkstra's algorithm, using a coupling algorithm, figure 2*b* and *c*. The number of surface elements (*n_i_*) that belong to each outlet is calculated by

2.18 $$\begin{array}{c} n_{i}=f_{i}N_{\mathrm{Tot}}, \end{array}$$

with *N*_Tot_ the number of triangles of the uniform triangulated mesh.

The coupling algorithm ensures that the size of the perfusion territories is scaled based on the cubed radius of vessel that perfuses it, i.e. Murray's Law. After the coupling, any unassigned surface elements are assigned to the nearest cluster. The surface elements are assigned to the nearest outlet based on their separation on the surface. This is an iterative algorithm to account for the mismatch between the pial surface and the outlet. The outlet position is updated by taking the centre point of the assigned surface elements as a new guess. The new centre point is determined by minimizing the maximum separation between points.

Data from vessel-encoded arterial spin labelling (VEASL) is used to confine the sampling of the mesh to regions that belong to the same major cerebral vessel [32–34]. This is done to achieve a better match between the estimated area and area of the surface mesh. The pial surface mesh does not contain the complex folding of the brain nor does it contain well separated frontal and temporal lobes. By using VEASL data, the mismatch between the expected area and the area of the surface mesh can be limited to the major territories of the brain. The major territories of the brain are the left and right ACA, MCA and PCA, the cerebellum and the brainstem. Vessels perfusing the pial surface are confined to the equivalent regions in the VEASL data. The coupling algorithm tries to find the sets for which the sum of distances within the set of surface elements is minimal. Note that the cluster sizes are calculated beforehand using equation (2.18).

The root of the perfusion territories can be thought of as the mean position of the surface elements in that set. The surface is transformed into a graph and Dijkstra's distance algorithm is used, figure 2*b*. Euclidean distance can also be used if the surface is relatively flat. The pseudo code is given as follows:

(1) Determine the number of surface elements per outlet with equation (2.18).
(2) Project the outlet to the pial surface as an initial guess for the root.
(3) Find the closest set of elements to the root.
(4) Minimize the root–element distance within the clusters.
(5) Update the root.
(6) Iterate until convergence.
(7) Repeat for each major cerebral region.

The mean flow velocity at outlets of the blood flow model is distributed evenly across the relevant perfusion territory. Each triangle of the mesh receives a volume flow rate based on its fractional area such that the flow velocity, i.e. volume flow rate per area, is uniform within each perfusion territory and within the major region set by the VEASL data. The result of the coupling is shown in figure 4, error estimation in figure 5 and blood flow at the pial surface in figure 6.

## 3. Results and discussion

Extending patient-specific data on the anatomy of the CoW with high-resolution data from the BraVa sets and literature can solve some of the problems of missing vessels. We include the arteries starting at the heart such that we can capture the redirection of flow in the event of a stroke. This is often excluded from many models that look at stroke. For large vessel occlusions, it is likely that the redistribution of blood can have significant effects on perfusion. A more detailed study on this is in progress and will be reported elsewhere.

The extended patient vasculature, figure 1*d*, should contain sufficient detail to capture all relevant effects such as the redistribution of flow after a stroke and make it possible to model volume flow rates, pressure and other variables. Once the patient anatomy is extended to the smaller cerebral arteries, we can begin to map the flow in the arteries to the brain. Modelling the entire cerebral vasculature down to the microcirculation is computationally unfeasible and probably also not required for our purpose. Being able to switch to a more efficient model is, therefore, crucial. The 1D blood flow model used in this paper is commonly used, well studied and validated [16,23,25]. The outlets of the 1D blood flow model provide blood flow to the perfusion territories of the brain.

Statistically accurate vessel networks can be generated between the outlets of the BraVa sets and the coupling points at the pial surface (figure 1*c*) using constrained constructive optimization [35–37]. However, one of the underlying assumptions of such algorithms is that the radii at the terminal points are equal and their positions are randomly chosen within a target volume. Since the assumption here is that there is no seepage through the vessel wall, the volume flow rates at the outlets would all be equal for a symmetric bifurcating tree. Simple scaling laws, such as Murray's Law and a length-to-radius ratio, can be used to generate bifurcating trees, as the 1D blood flow model only depends on the length and the radius and not the position of the vessels.

Pulsatile blood flow was simulated in the anatomy of figure 1*d*, using parameters from table 2. The simulations were performed for seven heartbeats, after which the solution converged. Time-dependent volume flow rate and pressure along the anatomy were obtained, and from that both velocity and pressure pulsatility indices (PI) were computed.

**Figure 3.**
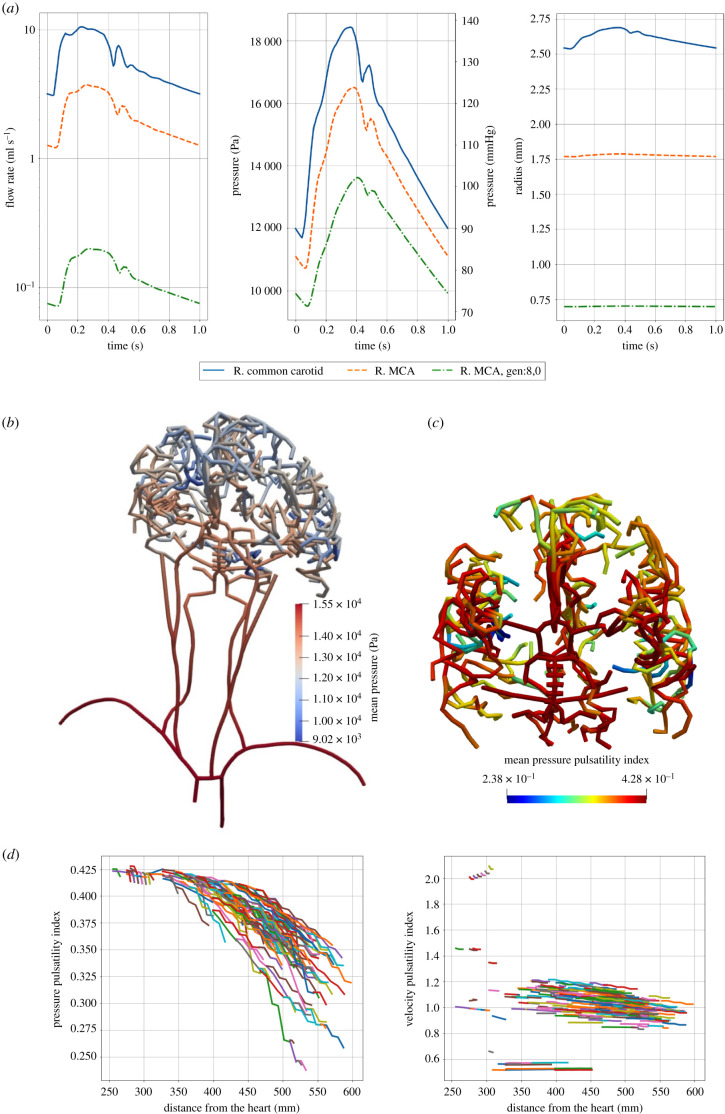
(*a*) Results from the 1D blood flow simulation for three different locations in the vasculature. The volume flow rate, pressure and radius over time are plotted. The locations are chosen based on their size. The chosen vessels were the right common carotid artery, the right MCA and one outlet vessel belonging to the right middle cerebral vasculature. (*b*) Mean pressure for the patient-specific vasculature calculated by the 1D blood flow model. Note that the vasculature shown here is mapped to a 3D anatomy that is the same for all patients and is used only for visualization; the length and radii are not to scale. (*c*) Pressure pulsatility index in the patient-specific vasculature of the CoW and downstream vessels. (*d*) Pressure and velocity pulsatility indices as a function of distance from the heart. Each line represents an individual vessel. Only vessels belonging to the CoW and beyond are shown.

The pressure and velocity pulsatility decrease as a function of distance from the heart for most vessels. Pulse pressure amplification is observed at the brachial arteries. Figure 3*a* shows the volume flow rate, pressure and radius of three vessels at different sizes, one large, one intermediate and one small artery. The chosen vessels were the right common carotid artery, the right MCA and one outlet vessel belonging to the right middle cerebral vasculature. The figure shows that the volume flow rate profile only changes in magnitude as the flow is split at every bifurcation. The pressure profile changes as the higher frequencies are damped. The vessel radius changes depending on the pressure and the properties of the vessel. The radii of the small cerebral arteries do not change much. The cerebral vessels are much stiffer than the large arteries and the pressure is lower further away from the heart, figure 3*b* [38]. The cerebral vessels beyond the CoW are similar in size; their Young's modulus is assumed to be similar and is set to the same value as their main branch of 1.6 MPa.

Figure 3*c* shows the pressure PI in the cerebral vasculature. Figure 3*d* shows the pressure and velocity PIs as a function of distance from the heart. Measurements of the velocity pulsatility index of the right MCA are possible through transcranial Doppler ultrasound. The PI measured is often the velocity PI also known as Gosling's pulsatility index [39]. PI is thought to reflect the downstream vascular resistance and changes in PI are associated with various diseases [40,41].

Table 3 shows the velocity pulsatility indices for the MCA. Comparing the values found to those in the literature shows that the model overestimates the velocity PI [40,42–44]. Stiffness of the vessels plays an important role in the transmission of pressure and velocity pulsatility through the vasculature [44,45]. Obtaining patient-specific Young's moduli for every vessel is difficult and can explain some of the overestimation of the velocity PI in the model. The outlet resistance and choice of BraVa segmentation affect the pulsatility indices more than the Young's moduli. Pressure pulsatility index decreases more than the velocity pulsatility index as a function of distance from the heart. This is likely due to the dampening seen in the pressure profiles, figure 3*a*. This suggests that the pressure PI is a better measure of the downstream resistance than the velocity PI.

**Table 3.** Velocity pulsatility index of the MCA.

origin	mean (s.d.)
model (default anatomy)	1.02
model with patient data	1.08
[42]	0.754 (0.110) (women) 0.823 (0.154) (male)
[43]	0.74 (0.04)
[40]	0.86 (0.18)
[44]	0.92 (0.21) (women) 0.94 (0.19) (men)

The pressure and velocity pulsatility decrease as the radius of the vessel decreases, figure 3*c* and *d*. High frequencies are effectively decreased in magnitude. The pressure pulsatility index shows that there are two types of behaviour. The first type is the pulse pressure amplification in the brachial arteries. The second type is the slow decrease in pulsatility index as distance from the heart increases. Velocity pulsatility seems to reach a minimum at around one.

Tables 3 and 4 show the mean PI and mean volume flow rates for the model with and without patient segmentations used to personalize the model. The configuration of the CoW and the stroke itself will change the outcome such that we cannot compare the model output to values from the literature for healthy patients. Note that the model is not calibrated nor fitted to match the reference values. The pulsatility indices when compared with the reference value do seem to be too high but, without either calibration or sufficient patient data, this is not surprising. The configuration of the CoW plays a major role in cerebral blood flow, especially during a stroke [16,46]. Including more patient-specific parameters, such as length, radii, elasticity, etc., will affect the model results. However, these are often difficult or infeasible to obtain for stroke patients. Getting correlated high-quality data for stroke patients is difficult. This is something that we hope to address in future work.

**Table 4.** Volumetric flow rates (ml min^−1^) per vessel for the model with and without patient segmentations.

vessel	model (patient)	model (default)	relative difference (%)
ascending aorta	4.20 × 10^3^	4.20 × 10^3^	0.00
aortic arch I	3.53 × 10^3^	3.54 × 10^3^	−0.28
brachiocephalic	6.76 × 10^2^	6.61 × 10^2^	2.22
aortic arch II	3.15 × 10^3^	3.19 × 10^3^	−1.27
L. common carotid	3.80 × 10^2^	3.47 × 10^2^	8.68
R. common carotid	3.67 × 10^2^	3.57 × 10^2^	2.72
R. subclavian	3.05 × 10^2^	3.03 × 10^2^	0.66
thoracic aorta	2.84 × 10^3^	2.89 × 10^3^	−1.76
L. subclavian	3.05 × 10^2^	3.03 × 10^2^	0.66
L ext. carotid	8.64 × 10^1^	1.47 × 10^2^	−70.14
L int. carotid I	2.92 × 10^2^	1.98 × 10^2^	32.19
R int. carotid I	2.80 × 10^2^	2.09 × 10^2^	25.36
R ext. carotid	8.71 × 10^1^	1.48 × 10^2^	−69.92
R. vertebral	8.82 × 10^1^	8.20 × 10^1^	7.03
R. brachial	2.17 × 10^2^	2.21 × 10^2^	−1.84
L. brachial	2.17 × 10^2^	2.21 × 10^2^	−1.84
L. vertebral	8.83 × 10^1^	8.21 × 10^1^	7.02
L. PCoA	−8.35	1.30 × 10^1^	255.69
R. PCoA	5.65 × 10^1^	1.37 × 10^1^	75.75
basilar I	1.50 × 10^2^	1.16 × 10^2^	22.67
L. MCA	1.93 × 10^2^	1.22 × 10^2^	36.79
R. MCA	1.35 × 10^2^	1.25 × 10^2^	7.41
L. ACA, A1	1.08 × 10^2^	6.24 × 10^1^	42.22
R. ACA, A1	8.95 × 10^1^	6.92 × 10^1^	22.68
L. PCA, P1	−6.73 × 10^1^	−3.48 × 10^1^	48.29
R. PCA, P1	−5.65 × 10^1^	−3.27 × 10^1^	42.12
L. ACA, A2	1.08 × 10^2^	6.30 × 10^1^	41.67
R. ACA, A2	8.92 × 10^1^	6.96 × 10^1^	21.97
ACoA	—	1.28 × 10^−1^	—
L. PCA, P2	5.89 × 10^1^	4.82 × 10^1^	18.17
R. PCA, P2	1.13 × 10^2^	4.69 × 10^1^	58.50
R. SCA	1.20 × 10^1^	2.22 × 10^1^	−85.00
L. SCA	1.20 × 10^1^	2.22 × 10^1^	−85.00
R. AICA	6.46	1.18 × 10^1^	−82.66
L. AICA	6.46	1.18 × 10^1^	−82.66
basilar II	1.50 × 10^2^	1.15 × 10^2^	23.33
pontine I	2.06 × 10^−1^	3.76 × 10^−1^	−82.52
pontine II	2.05 × 10^−1^	3.74 × 10^−1^	−82.44
pontine III	2.04 × 10^−1^	3.73 × 10^−1^	−82.84
pontine IV	2.03 × 10^−1^	3.72 × 10^−1^	−83.25
pontine V	2.02 × 10^−1^	3.71 × 10^−1^	−83.66
pontine VI	2.02 × 10^−1^	3.70 × 10^−1^	−83.17
pontine VII	2.06 × 10^−1^	3.76 × 10^−1^	−82.52
pontine VIII	2.05 × 10^−1^	3.74 × 10^−1^	−82.44
pontine IX	2.04 × 10^−1^	3.73 × 10^−1^	−82.84
pontine X	2.03 × 10^−1^	3.72 × 10^−1^	−83.25
pontine XI	2.02 × 10^−1^	3.71 × 10^−1^	−83.66
pontine XII	2.02 × 10^−1^	3.70 × 10^−1^	−83.17
basilar III	1.49 × 10^2^	1.14 × 10^2^	23.49
basilar IV	−1.49 × 10^2^	−1.14 × 10^2^	23.49
basilar V	−1.48 × 10^2^	−1.13 × 10^2^	23.65
R. PICA	6.56	1.20 × 10^1^	−82.93
L. PICA	6.56	1.20 × 10^1^	−82.93
R. vertebral II	8.02 × 10^1^	6.89 × 10^1^	14.09
L. vertebral II	8.03 × 10^1^	6.90 × 10^1^	14.07

The coupling algorithm is able to consistently map the outlets of the blood flow model to the pial surface. The coupling method works with any pial surface mesh and a higher-resolution mesh will also increase the resolution of the perfusion territories. The choice of BraVa subject does not affect the blood flow variables before the CoW with the coefficient of variation not exceeding 0.005 for any vessel in table 1. However, there is variation in the location and size of the perfusion territories. This likely reflects the individuality of the cerebral vasculature. Computation times for the 1D blood flow simulation are of the order of 10 min while the coupling algorithm takes significantly more time and is of the order of 60 min. Computations are done on a standard desktop computer with an Intel core i7–7700 k running at 4.2 GHz with 16 GB RAM.

**Figure 4.**
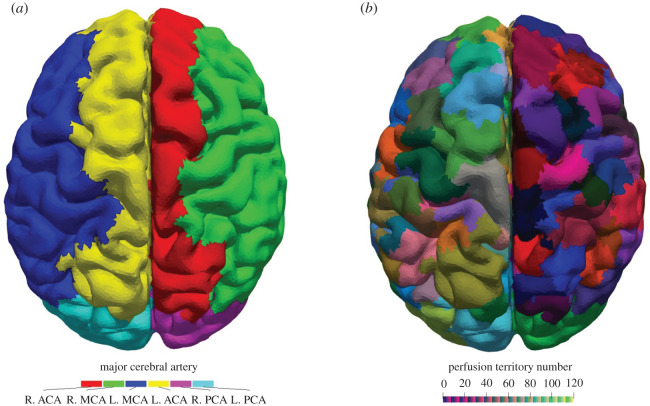
(*a*) The pial surface mapped by the coupling algorithm. The major region ID for each triangle of the pial surface mesh is shown. Note that this result is enforced by the algorithm. (*b*) The pial surface mapped by the coupling algorithm. Each triangle is coloured based on the cluster ID. Each coloured region corresponds to one outlet of the 1D blood flow model. Each cluster is one connected region and lies within its major cerebral vessel domain as shown in (*a*).

**Figure 5.**
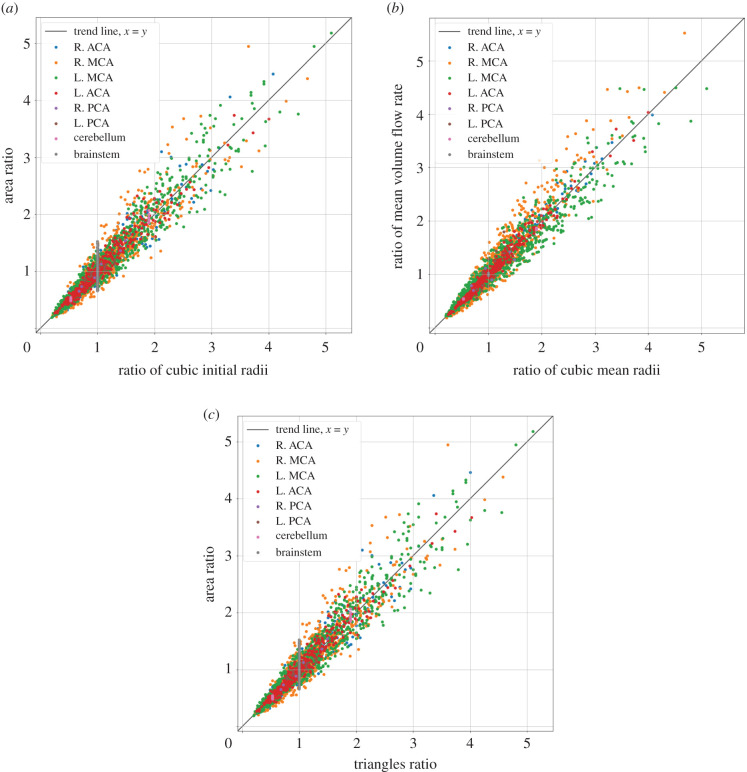
Relationships between perfusion territories within the same major region. The fractions are calculated as $r_{i}^{3}/r_{j}^{3},A_{i}/A_{j},Q_{i}/Q_{j},N_{i}/N_{j}$, where *i* and *j* denote the perfusion territories. (*a*) The fraction between cubed radii at the outlet and area on the pial surface. (*b*) The fraction between cubed radii at the outlet and the mean volume flow rate. (*c*) The fraction between the number of triangles of two clusters and their area. All figures show linear relationships.

Figure 4*a* shows the mapping of the pial surface based on the major artery from which the vessels originate. To date, only the perfusion territories for the major cerebral arteries are well established [15,47]. The major cerebral perfusion territories are enforced with the use of VEASL data. This is done to try to limit the error between the expected area and the area on the surface mesh. The surface mesh does not capture all the complex folding of the brain and a mismatch is expected. This lack of resolution is a serious limitation of the coupling algorithm. A downside of this is that the fractional areas, equation (2.17), are only strictly valid within each region, figure 5.

The coupling algorithm essentially maps the pial surface to the nearest vessel. The main benefit from the coupling algorithm is that it ensures that the perfusion territories are sized correctly, figure 5. Shown in figure 5 are ratios between various variables within regions of the brain. Every outlet is compared to every other outlet in a specific region of the brain. The ratios provide a way to compare the results of the model to the model assumptions as a form of verification. Figure 5*a* shows the relationship between the cubic radius and the area on the pial surface of the vessel outlets. Figure 5*b* shows the relationship between cubic radius and mean volume flow rates, see equation (2.16). Figure 5*c* shows the relation between the number of triangles and area on the surface mesh. The ratios display a linear relationship; any deviation from the linear relationship represents a deviation from the model assumptions. The deviations come from the fact that the resistance of the vasculature affects the flow and pressure at the outlet, and the surface segmentation is divided into a discrete number of triangles (numerical error).

The coupling method is graph-based and works with any geometry and distance metric. The coupling method uses equation (2.17) to estimate the fractional area of each outlet without the need to calculate flow rates. Once the outlets are mapped to the surface, the effect of different diseases, such as a stroke or stenosis, on blood flow can be modelled. The assumption is that the underlying vasculature does not change due to the disease. Different scaling laws can be applied by changing the calculation of fractional area, equation (2.17). Using a different scaling law affects the size and location of the perfusion territories. It would be interesting to see the effect of different scaling laws. Murray's Law is used in this paper as it seems to be a good fit for the cerebral vasculature [48].

A simple alternative to the coupling algorithm would be to map the surface to the nearest outlet. However, this can cause problems when some outlets are mapped to large areas of the surface and others are not mapped at all. The effect of differently sized radii at the ends of the vessels cannot be ignored. Another way to estimate perfusion territories is based on calculating streamlines [49]. However, this method is computationally expensive and can run into issues with large complicated geometries.

Changes in volume flow rate become negligible on the time scale of tissue oxygenation and cell death. We, therefore, argue that we can approximate the volume flow rates at the pial surface as steady-state flow. The mean volume flow rate over the region does not depend on details of the vasculature due to conservation of mass. The steady state only changes due to a major event in the vasculature such as a stroke. We, therefore, map the mean volume flow rate directly from the vessel outlet to its perfusion territory. One could go further and replace the entire blood flow model with a steady-state resistance model.

**Figure 6.**
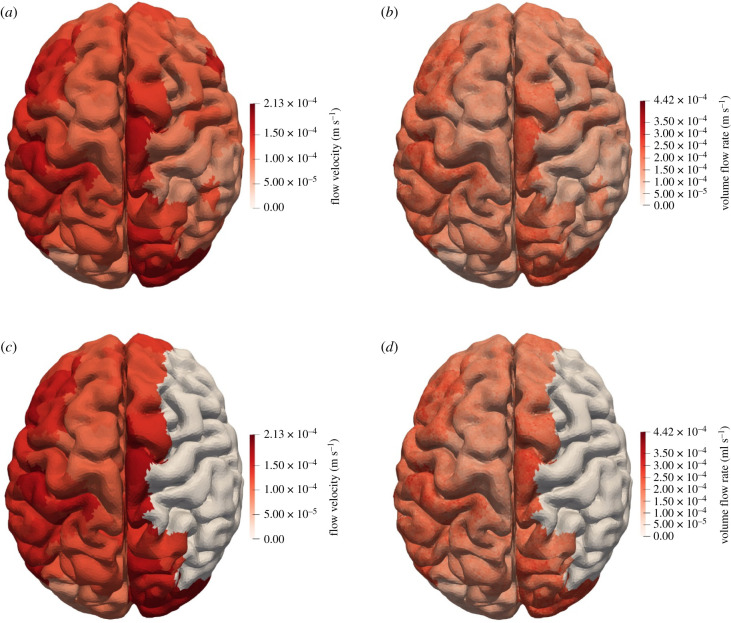
(*a*) Mean blood flow velocity at the pial surface for a healthy patient. The mean volume flow rate divided by the cluster area for each vessel outlet to the pial surface is shown. All values are within one order of magnitude of each other. (*b*) Volume flow rate on the mesh for a healthy patient. The volume flow rate at the outlet of the vessel is mapped to its cluster on the mesh. There are large differences between clusters, as the outlet radii can differ significantly. (*c*) Flow velocity at the pial surface for a patient with a stroke. There is a clot in the right MCA. Pressure after the clot is equal to that of the venous system and flow is zero. (*d*) Volume flow rate at the pial surface for a patient with a stroke. Any vessel originating from the right MCA has zero flow and their perfusion territories receive no blood.

The mapping of the pial surface to the outlets of the 1D blood flow model is shown in figure 4*b*. The volume flow rate at the outlets of the 1D blood flow model is distributed evenly across its perfusion territory. Figure 6*a* shows the mean blood flow velocity at the pial surface and figure 6*b* shows the mean volume blood flow rate at the pial surface. There are some differences in flow rate at the pial surface but all flow rates are within one order of magnitude. These differences are largely due to deviations from the assumed proportionality between radius and volume flow rate, equation (2.16) and figure 5*b*. The flow rates at the surface depend not just on the radius at the outlet but also on the resistances up to the outlets. The surface mesh also does not capture all the complexity of the human brain and affects the distribution of the flow across the pial surface, figure 5*a* and *c*. There is a trade-off between increasing the number of surface triangles to improve accuracy and computation time. Improving the quality of the surface mesh and improving the surface mapping as well as validation of the mapping will be addressed in future work. An extensive validation based on the MR CLEAN database is in progress. Infarcted volume prediction based on the model presented here coupled to a porous model shows promising agreement with CT scans [12].

A clot can be modelled by introducing a reflective boundary condition at the boundary of the flow. This is achieved by enforcing a zero pressure gradient, resulting in zero flow through the clot. The redistribution of flow after a stroke is included in the model. The flow to the affected region is redistributed to the other outlets. The brain receives a small portion of this flow; most goes to the other parts of the body. The brain receives only about 15% of cardiac output. A first approximation would, therefore, be that the brain receives 15% of the flow to the occluded region. Blood flow to the rest of the brain is not expected to be affected significantly. Figure 6*c* shows the effect of a clot in the right MCA on the velocity of flow at the pial surface. Figure 6*d* shows the effect of the same clot on the volume flow rate to the pial surface. The figures show that downstream of the clot there is no flow, leading to regions on the pial surface that lack flow. These are the areas where an infarct will start to form.

The framework presented here can be used to model blood flow to organs such as the brain. In addition, the coupling algorithm can be used to estimate perfusion territories for any organ as long as the appropriate distance metric is used. It is important to capture enough of the vasculature to be able to accurately predict pressure and flow rates. Obtaining correlated parameters as well as patient-specific segmentations is a major challenge for creating patient-specific simulations.

## 4. Conclusion

*In silico* trials can dramatically improve the process of development of medical devices, drugs and treatments. IST can potentially reduce the cost and duration of running a trial by reducing the need for patients and speeding up testing. The INSIST consortium [50] aims to create an IST for treatment of acute ischaemic stroke. The trial will include models of the patient population, arterial blood flow, tissue perfusion, metabolism, thrombosis, thrombolysis and thrombectomy. Here, the arterial blood flow model and a method for estimating perfusion territories are presented. Image-based patient-specific data on the anatomy of the CoW are combined with literature data and models for vessel anatomy not visible in the images to create an extended model for each patient. An acute ischaemic stroke can be modelled as the blockage of flow at any point in the vasculature. Modelling stroke and predicting infarct volume requires the coupling of multiple models on different scales. A method to couple patient-specific blood flow models to a tissue perfusion model is presented. Blood flow to the pial surface can be approximated as steady if we accept a small error due to the loss of the time-dependent behaviour. However, additional work remains to be done on the incorporation of feedback between the blood flow model and the tissue perfusion model.

## Endnotes

1 See www.insist-h2020.eu.

2 iCafe. © 2016–2018 University of Washington. Used with permission. iCafe was developed by the Yuan Lab at the University of Washington.

## Data accessibility

Patient-specific data are not available due to privacy regulations. The BraVa dataset is freely available from http://cng.gmu.edu/brava. The model code is available on GitHub at https://github.com/Rpadmos/Coupling1DBF.
